# Feasibility, acceptability and change in health following a telephone-based cognitive behaviour therapy intervention for patients with axial spondyloarthritis

**DOI:** 10.1093/rap/rkaa063

**Published:** 2020-11-17

**Authors:** Rebecca Pedley, Linda E Dean, Ernest Choy, Karl Gaffney, Tanzeel Ijaz, Lesley Kay, Karina Lovell, Christine Molloy, Kathryn Martin, Jonathan Packham, Stefan Siebert, Raj Sengupta, Gary J Macfarlane, Rosemary J Hollick

**Affiliations:** 1 Division of Nursing, Midwifery and Social Work, School of Health Sciences, Faculty of Biology, Medicine and Health, University of Manchester and Manchester Academic Health Science Centre, Manchester; 2 Aberdeen Centre for Arthritis and Musculoskeletal Health (Epidemiology Group), School of Medicine, Medical Sciences and Nutrition, University of Aberdeen, Aberdeen; 3 CREATE Centre, Section of Rheumatology, Division of Infection and Immunity, Cardiff University, Cardiff; 4 Department of Rheumatology, Norfolk and Norwich University Hospital NHS Foundation Trust, Norwich; 5 Hywel Dda University Health Board, Haverfordwest; 6 Rheumatology Department, The Newcastle upon Tyne Hospitals NHS Foundation Trust, Newcastle; 7 Haywood Rheumatology Centre, Stoke on Trent; 8 Division of Epidemiology and Public Health, University of Nottingham; 9 Institute of Infection, Immunity and Inflammation, University of Glasgow, Glasgow; 10 Royal National Hospital for Rheumatic Diseases, Royal United Hospitals NHS Foundation Trust; 11 Department of Pharmacy and Pharmacology, University of Bath, Bath, UK

**Keywords:** axial SpA, fibromyalgia, telephone-based cognitive behavioural therapy, feasibility

## Abstract

**Objective:**

The aim was to assess the feasibility and acceptability of a telephone-based cognitive behaviour therapy (tCBT) intervention for individuals with axial SpA (axSpA), with and without co-morbid FM, and to measure the change in patient-reported health outcomes.

**Methods:**

A convenience sample of individuals recruited from British Society for Rheumatology Biologics Registry for AS (BSRBR-AS) sites were offered a course of tCBT (framed as coaching). Patient-reported outcomes were measured at baseline and on course completion. Semi-structured qualitative interviews assessed intervention acceptability. Thematic analysis was informed by the theoretical framework of acceptability.

**Results:**

Forty-two participants attended for initial assessment. Those completing at least one tCBT session (*n* = 28) were younger, more likely to meet classification criteria for FM (57 *vs* 29%) and reported higher disease activity. Modest improvements were reported across a range of disease activity and wider health measures, with 62% of patients self-rating their health as improved (median 13 weeks post-intervention). Twenty-six participants were interviewed (including six who discontinued after initial assessment). tCBT was widely acceptable, offering a personalized approach. Despite low or unclear expectations, participants described improved sleep and psychological well-being and gained new skills to support self-management. Reasons for non-uptake of tCBT centred on lack of perceived need and fit with individual value systems. Many felt that tCBT would be most useful closer to diagnosis.

**Conclusion:**

Higher uptake among axSpA patients with co-morbid FM suggests that these individuals have additional needs. The findings are helpful in identifying patients most likely to engage with and benefit from tCBT and to maximize participation.

**Key messages**

- Higher uptake of telephone-based cognitive behaviour therapy among axial SpA patients meeting criteria for FM suggests that they have additional needs.
- Telephone-based cognitive behaviour therapy might be of most benefit close to diagnosis.
- Acceptability of telephone-based cognitive behaviour therapy is enhanced by framing the intervention as coaching.
- Despite low expectations, participants reported modest short-term improvements in health outcomes.

## Introduction

FM is a common co-morbidity in patients with axial SpA (axSpA). Compared with a 2–4% population prevalence of FM using ACR 1990 classification criteria, a recent meta-analysis estimated that the prevalence of FM in axSpA patients was 13% [1]. Within the British Society of Rheumatology Biologics Registry for AS (BSRBR-AS), one in five patients met 2011 research criteria for FM [2]. AxSpA patients with co-morbid FM have a higher disease activity and worse quality of life (QoL) than those without FM [2–6]. Although meeting FM criteria is partly a reflection of high disease activity, it is specifically a high somatic symptom burden that primarily predicts an attenuated response to TNFi therapy [7, 8].

Effective strategies are needed to manage both inflammatory disease and co-morbid FM. Cognitive behaviour therapy (CBT) improves coping with pain and depressed mood [9] in FM, and the benefits are sustained over time. Our recent randomized controlled trial of telephone-delivered CBT (tCBT) and a community exercise programme for chronic widespread pain found clinically significant improvements in patient-reported health outcomes both at the end of a course of therapy [10] and 2 years later [11]. tCBT has also been shown to be cost effective [11], acceptable [12] and with improved adherence compared with face-to-face delivery [13].

However, whether the benefit of such therapy is specific to (or greatest in) patients with FM and axSpA or is more generally beneficial to patients with axSpA only is not known. Lack of engagement with CBT-based interventions is also problematic. Even when participants have agreed to take part in studies that include a CBT component, around one-third of patients do not engage [10]. Qualitative studies, exploring experiences of tCBT for the treatment or prevention of chronic widespread pain, have included only participants with experience of the intervention, omitting those who did not take up tCBT [12, 14]. The reasons for lack of uptake therefore remain unclear. Understanding acceptability is important to encourage recipients to engage fully with and benefit from new interventions [15].

The aim of this study was to assess the feasibility, acceptability and change in patient-reported health measures after a 6-week tCBT intervention for axSpA patients, with and without co-morbid FM. We also examined reasons for non-uptake and discontinuation of the intervention.

## Methods

### Participants and procedures

The Fibromyalgia Optimal Management for patients with Axial Spondyloarthritis (FOMAxS) study examined the co-occurrence of axSpA and FM in persons who met the Assessment of SpondyloArthritis international Society (ASAS) criteria for axSpA. Participants from the BSRBR-AS study who had given consent to be contacted regarding other studies were eligible. In addition, patients from other centres who met BSRBR-AS eligibility criteria were eligible.

A convenience sample of FOMAxS participants were invited to take part in a tCBT intervention. Given that 30 participants is thought to represent an adequate sample size for an acceptability study [16], we aimed to recruit ≥15 participants who met the 2011 research criteria for FM and 15 who did not. Based on the tCBT intervention in the Managing Unexplained Symptoms In primary Care: Involving traditional and Accessible New approaches (MUSICIAN) trial [10], the study intervention included an initial assessment (45–60 min), followed by six weekly one-to-one sessions (30 min each). This was delivered remotely by a psychological well-being practitioner (PWP) and supported by a CBT-based manual. PWPs undertake accredited training in improving access to psychological therapies (in this case, a postgraduate certificate for advanced interventions in mental health) and support people with common mental health problems within the NHS. The PWP (C.M.) had ∼4 years of experience in delivering CBT-based treatments. Although treatment fidelity was not assessed formally, adherence to the overall approach was ensured through training and clinical supervision from K.L., author of the coaching manual.

### Intervention content

The intervention was described to participants as coaching sessions, after feedback from a National Axial Spondyloarthritis stakeholder consensus meeting. Patients felt very strongly that the word therapy had a number of negative connotations within society, whereas the term coaching implied a more positive, active self-management approach that was more empowering. Individuals stressed their preference for a flexible approach and multiple tools (a tool kit) to help self-manage their axSpA and support them to react/adapt and manage what the day brings; every day, disease or not. Patients felt that the concept of coaching fitted well with this. The FOMAxS intervention was personalized, and consequently, the cognitive behavioural and behavioural change techniques (BCTs) used varied depending on the participant’s goal(s). However, BCTs were used generally throughout the sessions to enable the participant to gain the most benefit from the intervention and the activities they carried out between sessions. This included setting goals, planning how to achieve these goals, self-monitoring and reviewing progress towards goals, and modifying or setting new goals after review.

A list of BCTs used across the intervention using the taxonomy of techniques from Michie *et al.* [17] is provided in Supplementary Table S1, available at *Rheumatology Advances in Practice* online, and a Template for Intervention Description and Replication TIDieR checklist [18] in Supplementary Table S2, available at *Rheumatology Advances in Practice* online.

### Patient-reported outcomes

Baseline and follow-up questionnaires self-administered before commencing the course and after completion included sociodemographic factors and lifestyle factors (physical activity). Health-related quality of life was assessed by the AS Quality of Life index [ASQoL: scored from 0 (best) to 18 (worst)] [19], mental health by the Hospital Anxiety and Depression Scale [HADS: from 0 (best) to 21 (worst)] [20], fatigue by the Chalder Fatigue Scale [scored from 0 (best) to 11 (worst)] [21] and sleep disturbance by the Jenkins Sleep Evaluation Questionnaire (0–20) [22]. Spinal pain was assessed using a 10 cm visual analog scale, and Bath indices of disease activity (BASDAI) and function (BASFI) [scored from 0 (best) to 10 (worst)] [23, 24]. The 2011 research criteria for FM provided both widespread pain index and symptom severity scores (0–19 and 0–12, respectively, with higher scores indicating poorer states) [25]. Physical activity was assessed by the number of minutes spent walking (per day) and engaging in moderate or vigorous physical activity (per week) using questions included in UK Biobank. Follow-up questionnaires additionally included a single item in which participants rated the change in their health since the period before receiving tCBT (categorical item response: very much better, much better, a little better, no change, a little worse, much worse or very much worse).

### Acceptability and experiences of tCBT

All participants offered tCBT, including those who subsequently declined or discontinued treatment, were invited to take part in a qualitative telephone interview, up to the point of data saturation. The interviewer was not involved in delivery of the intervention. Interviews were conducted as soon as possible after the final coaching session or study withdrawal. Interviews explored expectations, perceived outcomes and perceptions of the intervention, and reasons for non-uptake or discontinuation. Interviews were recorded using an encrypted voice recorder and transcribed.

### Data analysis

Participants were described in terms of sociodemographic and patient-reported factors [proportions for categorical factors and median and inter-quartile range (IQR) for continuous factors]. Among those who received tCBT, the absolute changes in patient-reported measures (median time between baseline and follow-up questionnaires 13 weeks) were calculated (follow-up minus baseline score) and are presented as the median and IQR. Participants who rated the change in their health as being very much better, much better or a little better were considered to have improved since the period before receiving tCBT.

Interview transcripts were analysed using thematic analysis [26]. Inductive and deductive coding was undertaken using NVivo software (v.12 Pro), guided by the theoretical framework of acceptability (TFA) [15]. This incorporates seven constructs: ethicality, burden, coherence, self-efficacy, affective attitude, perceived effectiveness and opportunity costs (Table 1). Analysis was refined through regular meetings with a second researcher and wider discussions with the study team. Emerging themes were discussed with two patient groups convened by the National Axial Spondyloarthritis Society before finalization and write-up.

**Table 1:** Definitions of the seven constructs of the theoretical framework of acceptability (reproduced from Sekhon *et al.* [15])

Theoretical Framework of acceptability (TFA)	Definition
Ethicality	The extent to which the intervention has a good fit with an individual’s value system
Affective attitude	How an individual feels about the intervention, after taking part
Burden	The amount of effort that was required to participate in the intervention
Opportunity costs	The benefits, profits or values that were given up to engage in the intervention
Perceived effectiveness	Experienced effectiveness: the extent to which the intervention is perceived to have achieved its intended purpose
Self-efficacy	The participant's confidence that they can perform the behaviour(s) required to participate in the intervention
Intervention coherence	The extent to which the participant understands the intervention and how it works

The BSRBR-AS received ethical approval from the National Research Ethics Service Committee North East – County Durham and Tees Valley (reference 11/NE/0374). Ethical permission for the FOMAxS study was granted by the South East Scotland Research Ethics Committee 1 (18/SS/0020). Written consent was obtained from participants before any study-related procedures were conducted.

## Results

Forty-seven individuals were approached to participate in tCBT. Five were subsequently unable to be contacted to arrange initial assessment; therefore, 42 individuals received an initial tCBT assessment (via telephone). Fourteen individuals did not subsequently wish to proceed with a course of tCBT or reached an agreement with the coach that they did not require tCBT (hereafter termed non-engagers). Twenty-eight participants completed at least one tCBT session (hereafter termed engagers), of whom 17 (61%) participated in the full six sessions [median number of sessions 6 (IQR 3.5, 6)]. Engagers were younger than non-engagers [median age of 59 (IQR 49, 70) *vs* 69 years (51, 74)], with more co-morbid FM (57 *vs* 29%) and higher disease activity [BASDAI 5.6 (IQR 2.4, 7.0) *vs* 3.4 (1.2, 5.8)].

More than two-thirds of engagers were educated beyond secondary school, and approximately one-third were in full time employment. Forty-one per cent were female, with a median disease duration of 36 years (IQR 12, 48) (Table 2). At baseline, engagers reported moderate levels of spinal pain [median 5.0 (IQR 2.0, 7.8)], health-related quality of life [8.0 (3.0, 11.0)], fatigue [3.5 (1.5, 7.0)], depression [4.0 (2.5, 8.0)] and anxiety [9.0 (5.0, 12.5)] but poor disease activity, physical function and sleep disturbance scores [BASDAI: 5.6 (2.4, 7.0); BASFI: 5.1 (2.1, 7.2); Jenkins: 10.5 (5.5, 16.0)]. Median widespread pain and symptom severity scores for this group were 6.0 (2.0, 11.0) and 6.0 (4.0, 10.0), respectively.

**Table 2:** Baseline descriptors of those offered CBT and who attended at least one session (*n* = 28)

Baseline factors		Frequency	Percentage/median (IQR)
Age		28	59 (49, 70)
Gender	Female	11	40.7
	Male	16	59.3
Education	Secondary school	6	24.0
	Apprenticeship/college	10	40.0
	University/further education	9	36.0
Employment	Full-time/unpaid	9	34.6
	Part-time	5	19.2
	Retired	9	34.6
	Retired/unemployed (owing to health)	3	11.5
Prescribed a biologic DMARD?	Yes	13	48.2
	No	14	51.8
Disease duration^a^	Years	25	36 (12, 48)
Physical activity	Minutes of walking/day	25	60 (25, 120)
	Days with moderate physical activity/week	25	4 (2, 7)
	Days with vigorous physical activity/week	26	2 (0, 4)
Spinal pain VAS	0 (best)–10 (worst)	26	5.0 (2.0, 7.8)
Disease activity	BASDAI: 0 (best)–10 (worst)	26	5.6 (2.4, 7.0)
Functional impairment	BASFI: 0 (best)–10 (worst)	25	5.1 (2.1, 7.2)
Quality of life	ASQoL: 0 (best)–18 (worst)	23	8.0 (3.0, 11.0)
Fatigue	CFS: 0 (best)–11 (worst)	24	3.5 (1.5, 7.0)
Sleep disturbance	Jenkins: 0 (best)–20 (worst)	24	10.5 (5.5, 16.0)
Anxiety	HADS: 0 (best)–21 (worst)	24	9.0 (5.0, 12.5)
Depression	HADS: 0 (best)–21 (worst)	24	4.0 (2.5, 8.0)
Widespread pain index	0 (best)–19 (worst)	21	6.0 (2.0, 11.0)
Symptom severity score	0 (best)–12 (worst)	25	6.0 (4.0, 10.0)

a Years from symptom onset to screening visit.

ASQoL: AS Quality of Life Index; CBT: cognitive behavioural therapy; CFS: Chalder Fatigue Scale; HADS: Hospital Anxiety and Depression Scale; IQR: inter-quartile range; VAS: visual analog scale.

Sixty-two per cent of engager patients self-rated their health as improved (median 13 weeks post intervention; Table 3). There were no significant differences in outcome between those who met criteria for FM and those who did not; therefore, combined results are presented. Engagers generally reported a median one point reduction across a range of disease-specific and general health measures: spinal pain [median −1.0 (IQR −1.5, 0.0)], disease activity [−0.9 (−1.5, 0.2)], fatigue [−1.0 (−2.0, 0.0)], sleep disturbance [−1.0 (−3.0, 1.0)], anxiety levels [−1.0 (−3.0, 1.0)] and widespread pain index [−1.0 (−2.0, 0.0)]. Smaller median improvements were noted for both physical function and FM symptom severity [−0.3 (−0.7, 0.1) and −0.5 (−1.5, 1.0), respectively], and there was no median change in quality of life [0.0 (−2.0, 1.0)] or depression levels [0.0 (−2.0, 1.0)].

**Table 3:** Patient-reported outcome change among those offered CBT and attending at least one session (*n* = 22)

	Frequency	Percentage
**Self-report health change (collapsed)**		
Improved	13	61.9
Same	3	14.3
Worse	5	23.8
**Absolute change in:**	**Frequency**	**Median (IQR)**
Spinal pain VAS	20	−1.0 (−1.5, 0.0)
Disease activity (BASDAI)	20	−0.9 (−1.5, 0.2)
Functional impairment (BASFI)	19	−0.3 (−0.7, 0.1)
Quality of life (ASQoL)	15	0.0 (−2.0, 1.0)
Fatigue (CFS)	18	−1.0 (−2.0, 0.0)
Sleep disturbance (Jenkins)	18	−1.0 (−3.0, 1.0)
Anxiety (HADS)	18	−1.0 (−3.0, 1.0)
Depression (HADS)	18	0.0 (−2.0, 1.0)
Widespread pain index	13	−1.0 (−2.0, 0.0)
Symptom severity score	20	−0.5 (−1.5, 1.0)

ASQoL: AS Quality of Life Index; CFS: Chalder Fatigue Scale; HADS: Hospital Anxiety and Depression Scale; IQR: inter-quartile range; VAS: visual analogue scale.

### Acceptability and experiences of tCBT

Twenty-six participants consented to be interviewed, including 6 non-engagers and 20 engagers. Of these, 35% were female, with a median age of 63 years and disease duration of 37 years. Thirty-eight per cent met criteria for FM. Emergent themes included all but one construct of the TFA (opportunity costs), plus an additional emergent theme (perceived need).

### Perceived need: variability in perceived need influenced uptake of the intervention

A common reason for non-uptake of coaching was that symptoms or impacts of axSpA were perceived as manageable at the time of assessment. Access to good health care, supportive friends and family, keeping active and eating well supported self-management. After undertaking assessment, the following individual made a decision with his coach to discontinue:

I don’t think I can get any better than I am, put it that way, like, I’m okay, I’m up and running….

Patient 14, male, zero sessions.

Others dismissed ongoing symptoms as normal for their age:

…whether it’s down to just getting old or whether it’s down to the AS I really don’t know. I would always assume it’s with just getting older.

Patient 6, male, zero sessions.

Many felt that tCBT would have been most beneficial earlier in their condition, when they had struggled most to cope with their illness. Receiving tCBT many years after illness onset felt too late for some, either because they saw the illness as unmodifiable or because they already felt able to cope. The following participant described some benefits but felt there was nothing more to address after two sessions:

…I feel this programme’s been well set up, but it is something people who are just being diagnosed with AS should have, and not me as an old-stager who’s worked her way through it and sorted out what’s what.

Patient 8, female, two sessions.

Participants identified people who they perceived would be most likely to benefit from tCBT, such as those in more pain, people with mental health problems and those lacking social support:

…if I’d have been maybe in my mid-40s, completely housebound and depressed, I could see that the coaching might be of help. But I’m not.

Patient 10, male, zero sessions.

In some, cases however, participants who initially perceived themselves to have low need changed their opinion after further discussion about how coaching might be able to help:

…because obviously I knew I didn’t suffer that much with AS. I, kind of, wondered what we were going to talk about through the sessions, and it was only, I think it was only during that first session when we, sort of, really hit on the sleep side of things that things, sort of, clicked for me. I realized that’s good, I can probably help you and you can probably help me…

Patient 11, male, six sessions.

There was also an admission among some that even though the treatment would have been more helpful earlier in the course of their condition, it had still proved highly valuable:

…hit the target area before they’re diagnosed or when they’re first diagnosed, that’s the only thing I would … (change).… But having said that, if you look at the likes of me, if I hadn’t have been included in the study group, I would still be a grumpy old man who wasn’t getting proper sleep.….

Patient 9, male, six sessions.

### Ethicality: the extent to which the intervention has a good fit with an individual’s value system

Stoicism and a belief in self-sufficiency contributed to a perceived lack of need for tCBT and influenced decisions to take part:

I don’t feel that I am in that much of pain … discomfort that I want to discuss it with someone on the end of the phone, that it's going to help me in any way.… I just tend to deal with it myself. I’m probably old school … at the end of the day, I have still got to get up and go to work.

Patient 12, male, zero sessions.

In contrast, having an open attitude to managing axSpA facilitated engagement. Use of the term coaching rather than therapy helped some to overcome preconceived ideas about CBT:

…from somebody who’s always tried to work and, I guess, go through the pain and carry on life as normal, the idea of coaching appealed to me a lot more, because it sounded like something more practical. And less emotional, but it ended up being a bit of both, which is good.…

Patient 13, female, six sessions.

### Burden: amount of effort required to participate in the intervention

A telephone-based approach was viewed as convenient, reducing time, stress and physical exertion associated with attending appointments face to face. Engaging by telephone helped participants to feel more relaxed and anonymous than they would do in a face-to-face clinical setting:

…I can get in the house, sit on my chair. I’m in my own little surroundings, so I’m relaxed and talking to a perfect stranger on the phone about my health problems….

Patient 9, male, six sessions.

This was enhanced by the coach, regarded as calm and easy to talk to. Flexible scheduling of appointments enabled participants to fit sessions around other commitments. For most, weekly sessions were regular enough to maintain momentum while allowing time to carry out homework. The coach ensured that tasks were clear and achievable:

…she [coach] broke it down into the bite sizes. And so that was easy to absorb during the week, and that made the whole thing easier to think, oh yes, actually, that’s been quite good. I can keep that going.…

Patient 4, female, six sessions.

In contrast, one participant felt that the demands of tCBT were too great given other issues in their life.

### Coherence: the extent to which the participant understands the intervention and how it works

Many participants were unsure what tCBT would involve or whether it would help, but were pleasantly surprised to find it beneficial:

I don’t know how you could get it across to them that this really is good because, like I said, I thought it would just be solely talking about AS but it wasn’t. It was talking about me with AS.

Patient 1, female, six sessions.

A lack of understanding about how tCBT might help participants reduced acceptability. For example, one participant struggled to see the relevance of poor sleep to his condition and would have preferred support that he perceived would more clearly address his physical symptoms:

That was the only thing that was getting me down a bit, [coach] kept asking me about the sleep pattern and all this and that, and I thought it would have been more, say, something I can do to, you know, like exercise or whatever, you know. Why are they on about the sleep pattern? I don’t know.

Patient 7, male, five sessions.

However, most participants’ understanding of how tCBT worked developed through experience; seeing the value of having someone to help them develop new ways of managing their condition and the ability to look at it in a different light.

### Self-efficacy: the participant's confidence that they can perform the behaviour(s) required to participate in the intervention

A number of features of tCBT supported participation and increased confidence. The quality of the working relationship with the coach was important. The ability of the coach to listen, be non-judgmental and respectful, provide encouragement and reassurance and demonstrate flexibility in adapting approaches according to individual needs, preferences and context were important.

The anonymity of telephone delivery enhanced the ability of participants to be open and honest:

…speaking to someone about things that are quite personal but having never met them was initially a bit disconcerting, but then I felt quite safe, like, well she doesn’t even know what I look like, she doesn’t even know where I am right now, I can say whatever I want.…

Patient 13, female, six sessions.

Although a number of theoretical concerns about tCBT were expressed (e.g. loss of visual cues impeding communication), in reality most reported being able to engage well with tCBT.

Most regarded the tCBT manual as informative and accessible. Many participants identified and used sections they could relate to, such as the case studies. However, some found reading the manual a struggle (preferring contact with the coach). Suggested improvements included better illustrations and greater diversity in the case studies, particularly in gender balance.

Occasionally, other physical health problems reduced the ability of participants to engage. Participants expressed regret that symptoms such as breathlessness and stomach problems made it difficult for them to engage in the exercises that they wanted to do, thus restricting their physical activity.

### Affective attitude: how an individual feels about the intervention after taking part

Participants expressed positive views about tCBT, which offered more time and opportunity to get to the nitty gritty compared with usual medical appointments:

…when I go to hospital for my annual check-ups to see the consultant, have them measure, look at my flexibility, that sort of thing, and don’t really get nothing out of it, if that makes sense. Thanks for coming, you’re still quite flexible, you’re still fairly young, it’s not really giving you any problems in your life, we’ll see you in a year. Whereas at least with [coach name] it was talking around it all, around how it makes me feel outside of work, how it makes me feel at work and then probably what help was there…

Patient 2, male, six sessions.

Participants valued the fact that tCBT took a holistic approach:

Your body, yes, you can manage that. But it's your mind that has to cope with it all. And to have a pathway open for you, as I did with [coach name], I think has made such a tremendous difference…

Patient 5, female, six sessions.

Most participants felt that the number, spacing and duration of tCBT sessions was sufficient. Those with lower perceived need sometimes found that fewer than six sessions were required, whereas a small number felt they might have benefitted from more sessions. Some suggested that booster sessions would be helpful to improve confidence and to check that they were managing to implement the tools/skills learned.

### Perceived effectiveness: the extent to which the intervention is perceived to have achieved its intended purpose

Most felt that tCBT brought significant benefits to their health, well-being and quality of life. Only a small number felt that nothing had changed. Setting and reviewing goals helped to motivate and overcome barriers to behaviour change:

…we can all put barriers in front of ourselves, can’t we, around why we can’t do it or why we decide not to do it. So that was quite good, having someone give me a bit of pressure around the goals I was setting and why I wasn’t doing them…

Patient 2, male, six sessions.

The ability of participants to self-manage their axSpA reportedly improved in a number of different ways; pacing of activities, encouraging practical steps to manage symptoms (e.g. purchasing new pillows, driving mirrors) and greater confidence about advocating for their needs in the workplace (e.g. speaking to their manager about reducing repetitive tasks). Improvements to mood and motivation and reduced guilt, worry and irritability were commonly reported:

…it’s definitely more mentally helpful … at the beginning I felt a bit more alone, a little bit trapped. And, I think, part of the guilt of talking about it or saying something is knowing that other people have a lot going on in their lives. So, speaking to [coach name] was very selfish time.…

Patient 13, female, six sessions.

Participants also reported that tCBT helped to facilitate changes to broader aspects of health and well-being, such as losing weight, improving diet, social functioning and relationships, by developing knowledge, skills and coping mechanisms that they continued to use beyond the sessions:

…it’s been fantastic, because now, instead of sitting here, thinking, right, and getting stressed out, I sort of can help myself a bit.

Patient 3, female, six sessions.

## Discussion

Individuals with axSpA, with and without FM, who participated in a 6-week tCBT programme reported modest, although not clinically significant, improvements across disease-specific and more general health measures ∼3 months after starting the programme. Those without co-morbid FM were less likely to engage with tCBT. Non-engagers reported low perceived need for tCBT and stoicism, and might have determined accurately that they had no need for it. In contrast, other participants, including some who were initially sceptical or unclear at the outset regarding the potential benefits of tCBT, subsequently found it useful and relevant. Among engagers, tCBT was widely acceptable, providing a holistic approach that participants thought was unavailable elsewhere, and provided skills and resources that promoted self-management. However, many participants felt that tCBT would have been most useful closer to diagnosis.

Interviews with a diverse sample of individuals (age, gender, presence or absence of FM, number of tCBT sessions completed), in addition to the inclusion of participants who did not take up tCBT, ensured that the dataset was not restricted to the views of individuals who found tCBT acceptable; the exclusion of those discontinuing tCBT is a limitation of previous acceptability studies [12].

However, our study has some limitations. The main aim of this study was to examine the feasibility and acceptability of a tCBT intervention, rather than to measure the effectiveness of treatment. We did not examine whether or not reported health benefits were maintained or increased over time, and formal evaluation of effectiveness will require a randomized controlled trial. Although treatment effectiveness was not the focus of this study, it is important in shaping experiences of treatment and acceptability. Yet despite initial scepticism and lack of clarity about its potential effectiveness, and modest short-term improvements in health outcomes, most participants still found the intervention useful, relevant and acceptable.

The intervention was delivered by a single coach experienced in delivering tCBT and who was employed specifically to deliver tCBT as part of the study. Perceptions of tCBT might differ in routine clinical settings. Telephone-based intervention and interviews might have presented a barrier to participation for those with communication difficulties. However, in light of the current COVID-19 pandemic, tCBT provides a safe and accessible means of intervention delivery.

The intervention was offered to everyone, and it is therefore possible that some participants received a treatment they did not need. However, there is no strong evidence base to predict precisely who benefits most from CBT. There was higher uptake of tCBT among those axSpA patients meeting criteria for FM, suggesting that these individuals have additional needs. Participants predominantly had established as opposed to early disease, and our findings suggest that tCBT might be more beneficial closer to diagnosis. Likewise, engagers had higher disease activity than non-engagers, suggesting that targeting individuals with moderate/high disease activity might improve engagement. However, a number of individuals with low perceived need at the outset subsequently found it beneficial as their understanding of the intervention developed; previously unmet needs were identified, and unanticipated benefits emerged.

Individuals with and without co-morbid FM reported modest improvements in health measures after tCBT. Although these modest benefits were not clinically significant, they are consistent with the benefits of tCBT reported elsewhere, which have been shown to increase with time and translate into longer-term improvements in quality of life and cost-effectiveness [11].

Interestingly, those receiving tCBT reported higher anxiety scores compared with depression scores at baseline, and many participants recalled heightened anxiety, especially around the time of initial diagnosis. axSpA patients with anxiety and depression report poorer quality of life and global health, greater fatigue and higher disease activity [27]. CBT-informed approaches have proven efficacy in the treatment of anxiety disorders [28] and might be particularly helpful for individuals with prominent anxiety. Our recent studies evaluating the acceptability of tCBT for the those with chronic widespread pain also found tCBT to be helpful, despite some initial scepticism about its benefits and lack of understanding of how a psychological intervention could improve a physical condition [12, 14]. Stoicism and self-reliance have been shown to have a negative influence on the attitudes of older adults to psychotherapy [29] and might have been more prevalent in this older study group with long-standing disease.

The TFA [15] sensitized the analysis to important aspects of acceptability. Emergent themes mapped onto a number of constructs of the framework (ethicality, burden, coherence, self-efficacy, affective attitude and perceived effectiveness). However, perceived need emerged as a separate construct, shaping acceptability of the tCBT intervention in a number of ways; influencing initial decisions to engage with the intervention and changing over time as participants’ understanding of the intervention developed, unmet needs were identified and previously unanticipated benefits emerged. Future studies should consider whether perceived need emerges in evaluations of acceptability across other conditions, suggesting a need to incorporate it as a construct within the TFA.

Our study findings are helpful in identifying patients who might benefit most from tCBT and informing approaches to improve engagement. tCBT might be of greater benefit to those more recently diagnosed with axSpA who have particular information needs [30]. Such patients are also more likely to be younger and of working age. We have previously shown that the ability to participate in work is important for people with axSpA [31], and future studies should consider the cost–benefit more widely in terms of gains in ability to work.

Reasons for non-uptake of tCBT centred on a lack of perceived need and lack of fit with their value system, rather than dissatisfaction with other aspects of the intervention. Our patient partners felt that framing the tCBT intervention as coaching rather than therapy conveyed a more positive, active self-management approach that might also be helpful to support self-management in other rheumatic diseases and in long-term conditions more broadly. Likewise, creating resources (such as the manual) and providing participants with brief case studies that showcase the array of difficulties tCBT can address might help to overcome unhelpful preconceptions and promote engagement. Flexibility in session delivery and the provision of booster sessions might facilitate ongoing engagement and support for self-management.

In conclusion, this study provides promising evidence of the acceptability and benefit of tCBT for patients with axSpA. Future testing to assess the clinical effectiveness and cost-effectiveness of tCBT in axSpA and other rheumatic diseases should draw on learning from this study, in particular how best to identify and target individuals most likely to engage and benefit and how to maximize participation.

## Supplementary Material

rkaa063_Supplementary_Data
