# Supplementary material for: Feasibility, acceptability and change in health following a telephone-based cognitive behaviour therapy intervention for patients with axial spondyloarthritis
Source: Rheumatol Adv Pract. 2020 Nov 17;5(2):rkaa063. doi: 10.1093/rap/rkaa063 (PMC8248414; doi:10.1093/rap/rkaa063)
Supplement: rkaa063_Supplementary_Data [file rkaa063_supplementary_data.zip › RAP 20-084.R1 Table S1 - accepted.docx]

**Supplementary Table S1. FOMAxS taxonomy table of behavioural change techniques**

| **No.** | **Label** | **Definition** |
| --- | --- | --- |
| **1. Goals and planning** | |  |
| **1.1** | ***Goal setting (behaviour)*** | Set or agree on a goal defined in terms of the behaviour to be achieved. |
| **1.2** | ***Problem solving*** | Analyse, or prompt the person to analyse, factors influencing the behaviour and generate or select strategies that include overcoming barriers and/or increasing facilitators (includes ‘Relapse Prevention*’ and ‘*Coping Planning*’*). |
| **1.3** | ***Goal setting (outcome)*** | Set or agree on a goal defined in terms of a positive **outcome** of wanted behaviour. |
| **1.4** | ***Action planning*** | Prompt detailed planning of performance of the behaviour (must include at least one of context, frequency, duration and intensity). Context may be environmental (physical or social) or internal (physical, emotional or cognitive). |
| **1.5** | ***Review behaviour goal(s)*** | Review behaviour goal(s) jointly with the person and consider modifying goal(s) or behaviour change strategy in light of achievement. This may lead to re-setting the same goal, a small change in that goal or setting a new goal instead of (or in addition to) the first, or no change. |
| **1.6** | ***Discrepancy between current behaviour and goal*** | Draw attention to discrepancies between a person’s current behaviour (in terms of the *form, frequency, duration, or intensity* of that behaviour) and the person’s previously set outcome goals, behavioural goals or action plans (goes beyond self-monitoring of behaviour). |
| **1.7** | ***Review outcome goal(s)*** | Review outcome goal(s) jointly with the person and consider modifying goal(s) in light of achievement. This may lead to re-setting the same goal, a small change in that goal or setting a new goal instead of, or in addition to the first goal. |
| **2.3** | ***Self-monitoring of behaviour*** | Establish a method for the person to monitor and record their behavior(s) as part of a behavior change strategy. |
| **2.4** | ***Self-monitoring of outcome(s) of behaviour*** | Establish a method for the person to monitor and record the **outcome(s)** of their behaviour as part of a behaviour change strategy. |
| **3.1** | ***Social support (unspecified)*** | Advise on, arrange or provide social support *(e.g. from friends, relatives, colleagues,’ buddies’ or staff)* or non-contingent praise or reward for performance of the behaviour*.* It includes encouragement and counselling, but only when it is directed at the **behaviour.** |
| **3.2** | ***Social support (practical)*** | Advise on, arrange, or provide **practical** help *(e.g. from friends, relatives, colleagues, ‘buddies’ or staff)* for performance of the behaviour. |
| **3.3** | ***Social support (emotional)*** | Advise on, arrange, or provide **emotional** social support *(e.g. from friends, relatives, colleagues, ‘buddies’ or staff)* for performance of the behaviour. |
| **4.1** | ***Instruction on how to perform a behaviour*** | Advise or agree on how to perform the behaviour (includes ‘**Skills training**’). |
| **4.2** | ***Information about antecedents*** | Provide information about antecedents (*e.g. social and environmental situations and events, emotions, cognitions)* that reliably predict performance of the behaviour. |
| **4.3** | ***Re-attribution*** | Elicit perceived causes of behaviour and suggest alternative explanations *(e.g. external or internal and stable or unstable).* |
| **4.4** | ***Behavioural experiments*** | Advise on how to identify and test hypotheses about the behaviour, its causes and consequences, by collecting and interpreting data. |
| **5.1** | ***Information about health consequences*** | Provide information (e.g. written, verbal, visual) about health consequences of performing the behaviour. |
| **5.4** | ***Monitoring of emotional consequences*** | Prompt assessment of **feelings** after attempts at performing the behaviour. |
| **5.6?** | ***Information about emotional consequences*** | Provide information (e.g. written, verbal, visual) about emotional consequences of performing the behaviour. |
| **7.1** | ***Prompts/cues*** | Introduce or define environmental or social stimulus with the purpose of prompting or cueing the behaviour. The prompt or cue would normally occur at the time or place of performance. |
| **8.2** | ***Behaviour substitution*** | Prompt substitution of the unwanted behaviour with a wanted or neutral behaviour. |
| **8.3** | ***Habit formation*** | Prompt rehearsal and repetition of the behaviour in the same context repeatedly so that the context elicits the behavior. |
| **8.4** | ***Habit reversal*** | Prompt rehearsal and repetition of an alternative behavior to **replace** an unwanted habitual behavior. |
| **8.6** | ***Generalisation of a target behavior*** | Advise to perform the wanted behaviour, which is already performed in a particular situation, in another situation. |
| **8.7** | ***Graded tasks*** | Set easy-to-perform tasks, making them increasingly difficult, but achievable, until behavior is performed. |
| **9.1** | ***Credible source*** | Present verbal or visual communication from a credible source **in favour of or against the behavior.** |
| **9.2** | ***Pros and cons*** | Advise the person to identify and compare reasons for wanting (pros) and not wanting to (cons) change the behavior. |
| **9.3** | ***Comparative imagining of future outcomes*** | Prompt or advise the imagining and comparing of future outcomes of changed versus unchanged behaviour. |
| **10.9** | ***Self-reward*** | Prompt self-praise or self-reward if and only if there ***has been*** effort and/or progress in performing the behavior. |
| **11.1** | ***Pharmacological support*** | Provide, or encourage the use of or adherence to, drugs to facilitate behavior change. |
| **11.2** | ***Reduce negative emotions ^b^*** | Advise on ways of reducing negative emotions to facilitate performance of the behavior (includes ‘**Stress Management**’). |
| **11.3** | ***Conserving mental resources*** | Advise on ways of minimising demands on mental resources to facilitate behavior change. |
| **12.1** | ***Restructuring the physical environment*** | Change, or advise to change the **physical** environment in order to facilitate performance of the wanted behavior or create barriers to the unwanted behavior (other than prompts/cues, rewards and punishments). |
| **12.2** | ***Restructuring the social environment*** | Change, or advise to change the **social** environment in order to facilitate performance of the wanted behavior or create barriers to the unwanted behavior (other than prompts/cues, rewards and punishments). |
| **12.3** | ***Avoidance/reducing exposure to cues for the behavior*** | Advise on how to avoid exposure to specific social and contextual/physical cues for the behavior, including changing daily or weekly routines. |
| **12.6** | ***Body changes*** | Alter body structure, functioning or support **directly** to facilitate behavior change. |
| **13.2** | ***Framing/reframing*** | Suggest the deliberate adoption of a perspective or new perspective on behavior (e.g. its purpose) in order to change cognitions or emotions about performing the behavior (includes ‘**Cognitive structuring**’). |
| **15.3** | ***Focus on past success*** | Advise to think about or list previous successes in performing the behavior (or parts of it). |
| **15.4** | ***Self-talk*** | Prompt positive self-talk (aloud or silently) before and during the behavior. |

# FOMAxS: Fibromyalgia Optimal Management for patients with axial Spondyloarthritis
